# DLIGAND2: an improved knowledge-based energy function for protein–ligand interactions using the distance-scaled, finite, ideal-gas reference state

**DOI:** 10.1186/s13321-019-0373-4

**Published:** 2019-08-07

**Authors:** Pin Chen, Yaobin Ke, Yutong Lu, Yunfei Du, Jiahui Li, Hui Yan, Huiying Zhao, Yaoqi Zhou, Yuedong Yang

**Affiliations:** 1 National Supercomputer Center in Guangzhou, School of Data and Computer Science, Sun Yat-sen University, 132 East Circle at University City, Guangzhou, 510006 China; 2 Sun Yat-sen Memorial Hospital, Sun Yat-sen University, Guangzhou, 510000 China; 3 Institute for Glycomics and School of Information and Communication Technology, Griffith University, Southport, QLD 4215 Australia

**Keywords:** Docking, Protein–ligand interaction, Knowledge-based energy function

## Abstract

Performance of structure-based molecular docking largely depends on the accuracy of scoring functions. One important type of scoring functions are knowledge-based potentials derived from known three-dimensional structures of proteins and/or protein–ligand complex structures. This study seeks to improve a knowledge-based protein–ligand potential based on a distance-scale finite ideal-gas reference (DFIRE) state (DLIGAND) by expanding the representation of protein atoms from 13 mol2 atom types to 167 residue-specific atom types, and employing a recently updated dataset containing 12,450 monomer protein chains for training. We found that the updated version DLIGAND2 has a consistent improvement over DLIGAND in predicting binding affinities for either native complex structures or docking-generated poses. More importantly, DLIGAND2 has a 52% increase over DLIGAND in enrichment factors in top 1% predictions based on the DUD-E decoy set, and consistently improves over Autodock Vina and other statistical energy functions in all three benchmark tests. We further found that DLIGAND2 outperforms empirical and machine-learning methods compared for virtual screening on new targets that are not homologous to the DUD-E training set. Given the best performance as a parameter-free statistical potential and among the best in all performance measures, DLIGAND2 should be useful for re-assessing the poses generated by docking software, or acting as one term in other scoring functions. The program is available at https://github.com/sysu-yanglab/DLIGAND2.

**Electronic supplementary material:**

The online version of this article (10.1186/s13321-019-0373-4) contains supplementary material, which is available to authorized users.

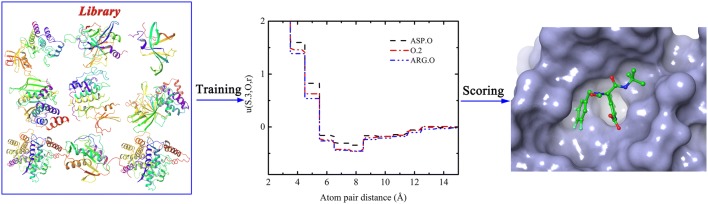

## Introduction

Structure-based molecular docking is one of the key components in computer-aided drug design [1–3]. Docking is a two-step process: conformational sampling of ligands bound to their receptors, followed by assessment of binding free energy between them. Due to advances in computing power and numerical algorithms, the success of docking is no longer restricted by inadequacy of conformational sampling but limited instead by the lack of a precise and reliable scoring function to evaluate the free energy of interactions between proteins and ligands [4]. Developing an accurate scoring function is challenging because molecular interaction is contributed by a delicate balance between several different types of interactions including van der Waals and columbic interactions in between, and interactions with solvent environment in addition to the difficulty in capturing entropic contributions [5, 6].

A wide variety of scoring functions has been developed to approximate energy functions. Based on the derivation ways, scoring functions are usually classified into physics-based methods, empirical scoring functions, knowledge-based potentials, and descriptor-based scoring functions [7]. Physics-based methods, widely employed in molecular dynamics simulation studies, are obtained by combing quantum mechanical calculations of small molecular fragments and empirical fitting to known experimental data. Some examples are linear interaction energy (LIE) [8, 9], linear response approximation (LRA) [10] and MM-PBSA/GBSA [11–13]. Since this type of methods require intensive computing time to perform kinetic integration for entropic effects, they are limited to assess a small number of compounds. Differently, virtual screening usually docked millions of molecules into a protein receptor to locate active compounds. Thus, the requirement of fast computation leads to the dominance of computationally efficient empirical scoring functions in docking as shown in the score-function assessment [5, 6]. Empirical scoring functions are based on a linear combination of various energetic terms to approximate binding free energy. Notable examples are ChemScore [14, 15], X-Score [16], Glide-Score [17, 18], and etc. Typically, the weight factors for individual energetic terms in an empirical scoring function are obtained by regression to achieve the highest correlation to experimental binding affinities (scoring power). More recently, machine learning methods have been used to combine energetic terms and/or employ protein–ligand distances for training. Examples are RF-Score [19], ID-Score [20], SVM-SP [21], and DrugVQA [22]. However, these scoring functions are often sensitive to docking poses and don’t perform well to separate decoys from true binding ligands in actual docking experiments [23]. Knowledge-based potentials (or statistical potentials) are derived from statistical analysis of known protein structures. A typical knowledge-based potential considers only the distances between atom pairs that allow efficient calculations. Different knowledge-based functions differ in how protein–ligand atom pair potentials and their reference states are defined. Examples are SmoG [24, 25], DrugScore [26], IT-Score [27, 28], and ASP [29]. Knowledge-based scoring functions are also used in combination with solvation and entropic terms to improve performance. Examples are DSX [30], SmoG2016 [31] and ITScore/SE [32].

Previously, a knowledge-based scoring function called DLIGAND [9] was developed based on the distance-scaled finite ideal-gas reference (DFIRE) state [33, 34], which has successfully been used for protein interactions with DNA [35], RNA [36], and carbohydrate [37] molecules. DLIGAND was developed by representing both protein and ligand atoms by a few mol2 atom types, and trained on a small set of 200 protein complex structures. Here, we developed DLIGAND2 by substituting 13 mol2 atom types by 167 residue-specific atom types for protein atoms and using a large protein structural dataset for training. We showed that DLIGAND2 not only significantly improves over DLIGAND but also has superior performance in separating true ligands from decoys in Database of Useful Decoys-Enhanced (DUD-E).

## Methods

### Scoring function

#### DLIGAND2 potential

We have used the same approach as the DLIGAND [38] to derive the distance-dependent interaction energy function between atomic pairs based on the distance-scale finite ideal-gas reference (DFIRE) state [33] as

1 $$ \bar{\mu } \left( {i,j,r} \right) = \left\{ {\begin{array}{*{20}c}    { - \eta RTln\frac{{N_{{obs}} \left( {i,j,r} \right)~~~}}{{\left( {\frac{r}{{r_{{cut}} }}} \right)^{\alpha } \left( {\frac{{\Delta r}}{{\Delta r_{{cut}} }}} \right)N_{{obs}} \left( {i,j,r_{{cut}} } \right)}},} & {r < r_{{cut}} }  \\    0,   \qquad      \quad\quad\quad\quad          \quad\quad\quad\quad     \quad & {r \ge r_{{cut}} }  \\   \end{array} } \right. $$

where R is the gas constant, T = 300 K, $\alpha$ = 1.61, *r*_*cut*_ = 15 Å, $\eta$ is a scaling factor simply set as 0.01/RT. N_obs_(i,j,r) is the number of atomic pair (i,j) within the spherical shell of distance r observed in a given structure database, and ∆r(∆r_cut_) is the bin width at r(r_cut_). A constant value of 0.5 Å was used for ∆r at all bins and ∆r_cut_ = ∆r. Here, we employed residue-specific atomic types for protein atoms that leads to 167 atomic types for protein atoms. This is different from DLIGAND, where both protein and ligand atoms were represented by mol2 atom types, and thus only 13 atom types were utilized for protein atoms.

We derived the protein–ligand interactions from protein structures because there is only a small number of non-redundant protein–ligand complex structures. From protein structures, we obtained the N_obs_ for the number of observed pairs between protein atoms, which are converted to protein–ligand interactions by mapping indices for protein atoms to 11 mol2 atom types (see Additional file 1: Table S1) and summing over all pairs that are mapped to the same mol2 atom type as

2 $$ N_{obs}^{{\prime }} \left( {i,k,r} \right) = \mathop \sum \limits_{j} N_{obs} \left( {i,j,r} \right)\delta \left( {map\left( j \right),k} \right) , $$

where *i* is protein atom type, $\delta \left( {map\left( j \right),k} \right)$ is 1 only when the protein atom type *j* is mapped to mol2 atom type *k*, otherwise 0. Based on the $N_{obs}^{{\prime }} \left( {i,k,r} \right)$, we can derive the potential function in the same manner as DFIRE. This design enables us to obtain the scoring function purely from protein atoms without requiring their binding partners, so we employed our recently collected 12,450 non-redundant protein monomer chains [39] to obtain a sufficient number of observations. This training set represents more than 60 times bigger than the dataset (195 complex structures) used for deriving DLIGAND. For ligand mol2 atom types not existed in proteins, they were mapped to the closest atom type, as detailed in Additional file 1: Table S1. We also adopted the low-count correction according to Bayesian statistics as the previous study [40].

### Benchmark datasets

Four benchmark datasets were employed to evaluate DLIGAND2. The first dataset is CASF-2013 [5, 6], a widely used benchmark containing 195 representative protein–ligand complexes. This benchmark has been used to test the accuracy of binding affinity prediction by using experimentally determined protein–ligand complex structures. The second dataset is the PDBbind refined set (version 2016) [41] of 4057 protein–ligand interaction pairs with experimentally measured binding affinity data. We generated protein–ligand complex structures by docking ligands onto their corresponding receptors respectively with eight docking packages, including AudoDock (version 4.2.6) [42], AutoDock Vina (version 1.1.2) [43], rDock (version 2013.1) [44], LeDock (version 1.0) [45], UCSF DOCK (version 6.8) [46], iDock (version 2013.1) [47], GalaxyDock (with BP2 Score) [48, 49], and iGEMDOCK (version 2.1) [50]. Docking ligands are confined to a 10 Å box enclosing the centroid of co-crystalized ligand. The maximum number of docking poses for each ligand was set to 10. After removing complexes failing to yield any complex structures in our selected docking programs, a collection of 4044 complexes remained for evaluation. The full list of 4044 complexes can be found in Additional file 2: Table S2. The scoring ability of functions were evaluated by the Pearson correlation coefficient (PCC) between the predicted and experimental values, as well as the root mean squared error (RMSE) after linear regression.

The ability of DLIGAND2 to perform virtual screening was also evalued on the DUD-E dataset [51]. There are 22, 886 active ligands binding with 102 targets, with an average of 224 ligands per target. For each target, the DUD-E database provides an abundant number of decoys (50 decoys for each active) that have similar physical–chemical properties but dissimilar two-dimensional (2D) topology. We employed the 3D structure of a target protein with the highest resolution in the protein data bank for docking. This is different from original DUD-E test where the 3D structure of the best performance was selected for each target [51]. For each pair of protein target and ligand compound, we employed Autodock Vina with default options to generate one pose, which are re-scored by 5 scoring functions (ΔvinaRF_20_, ID-Score, X-Score, DLIGAND, and DLIGAND2).

The accuracy of each scoring function was evaluated by the LogAUC and enrichment factor (EF).

As described in DUD-E Ref. [51] and our previous studies [52, 53], LogAUC takes the logarithm of x-axis in area under curve (AUC) to show more information on enrichment at a low false positive rate. We chose three regions of EF in top *x*% of the DUD-E dataset, where *x* equals to 1, 5 and 10 respectively.

3 $$ EF^{x\% } = \frac{{{\raise0.7ex\hbox{${N_{True}^{x\% } }$} \!\mathord{\left/ {\vphantom {{N_{True}^{x\% } } {N_{Selected}^{x\% } }}}\right.\kern-0pt} \!\lower0.7ex\hbox{${N_{Selected}^{x\% } }$}}}}{{{\raise0.7ex\hbox{${N_{Active} }$} \!\mathord{\left/ {\vphantom {{N_{Active} } {N_{Total} }}}\right.\kern-0pt} \!\lower0.7ex\hbox{${N_{Total} }$}}}} $$

where $N_{True}^{x\% }$, $N_{Selected}^{x\% }$, $N_{Selected}^{x\% }$ and $N_{Total}$ are the number of true positives, the number of selected candidates at top *x*% screened candidates, the number of active compounds, and the total number of compounds in the screened library, respectively.

For a fair comparison with the machine-learning-based scoring function (RF-Score-VS [54]) trained on the DUD-E dataset, we selected protein targets from the DEKOIS 2.0 benchmark [55] if it has sequence identity less than 95% to any protein in the DUD-E according to the BLAST [56]. Finally, 55 targets were kept and sorted by their sequence identity, as detailed in Additional file 3: Table S3.

## Results and discussion

### The DLIGAND2 potential

**Fig. 1:**
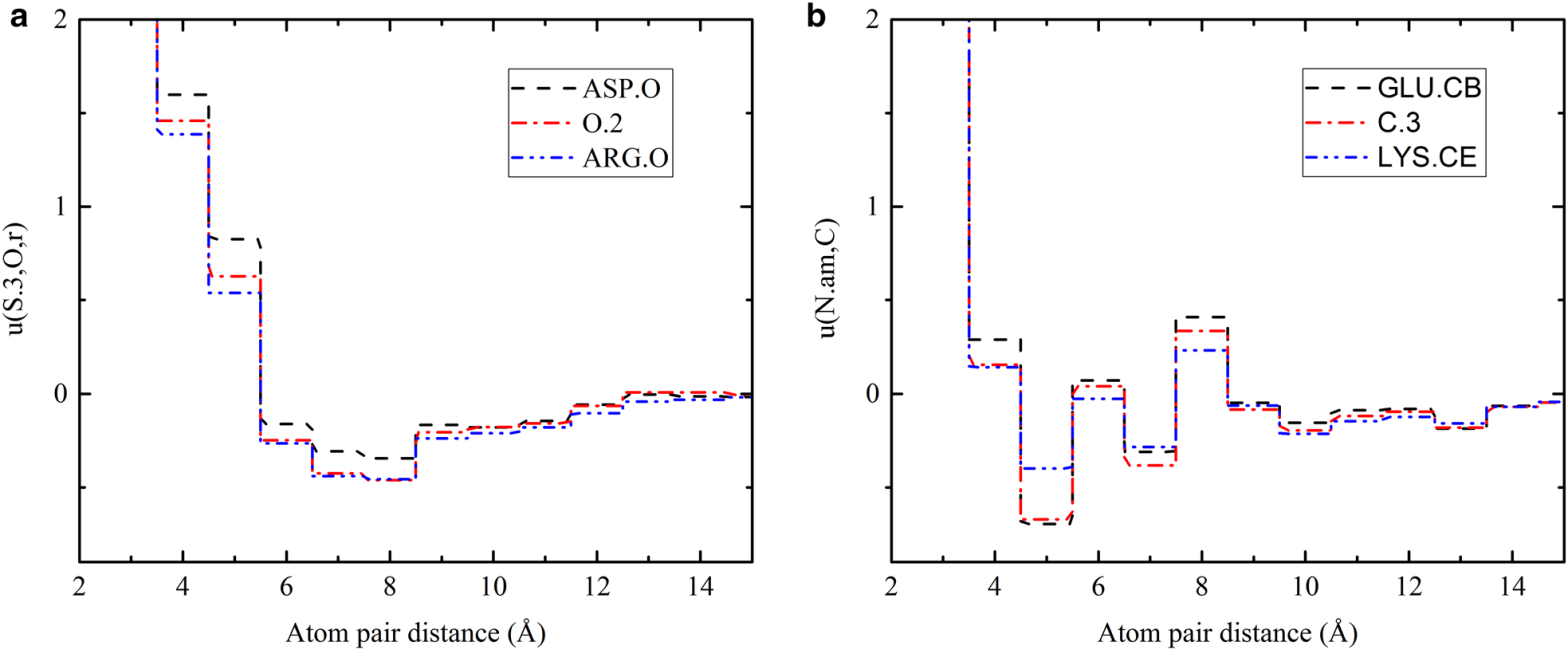
The atomic interaction potentials **a** between ligand type S.3 and main-chain O atom of ASP, or ARG in DLIGAND2, or their common mol2 atom type (O.2) by the DLIGAND, and **b** between ligand type N.am and atom CB of GLU, or CE of LYS, or their common mol2 type “C.3” by DLIGAND, as a function of distance

Different from the united mol2 atom type used by DLIGAND, the improved version DLIGAND2 has employed residue-specific types for protein atoms, which expanded atom types from 12 types to 169 atom types. Sufficient statistics for this larger number of atom types is ensured by using 12,450 protein chains for training. Residue-specific atom types enable the discrimination of the properties (e.g. partial charge) and surrounding environments of atoms. As shown in Fig. 1a, the potential energy between ligand atom S.3 and the main-chain O atom of ASP is significantly lower than between the atom and the main-chain O atom of ARG likely because S.3 atom has a weak but negative partial charge, which is repulsive to the negative charged ASP but attractive to the positive charged ARG residue. By comparison, DLIGAND provides an average potential over 20 amino acids. Significant differences also exist for interactions involving non-polar atoms. As shown in Fig. 1b, the CB atom of GLU and the CE atom of LYS belong to C.3 as defined in mol2, despite their very different electrostatic and steric environment. Their interactions with the ligand type N.am are very different when derived independently (DLIGAND2), and enclose the average energy function from DLIGAND.

### Evaluation results on CASF-2013 benchmark

#### Score power

**Fig. 2:**
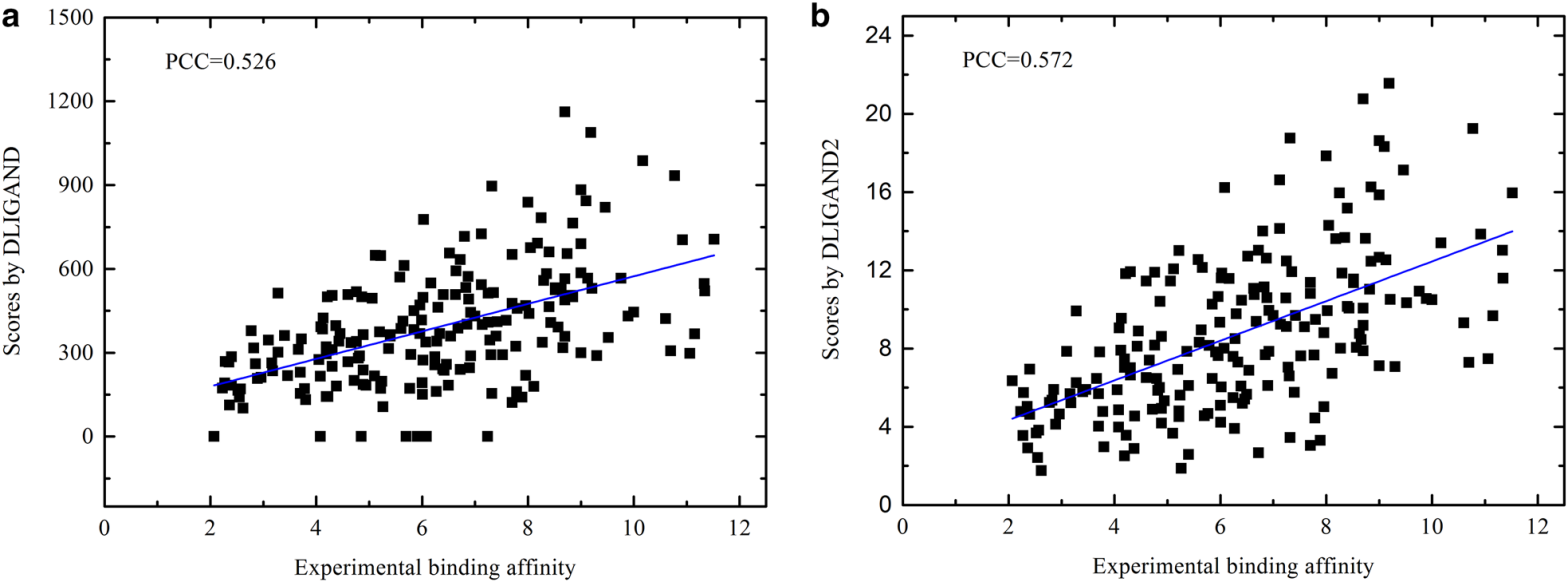
Comparison between theoretically predicted and experimentally measured protein–ligand binding free energies for 195 complexes on the CASF-2013 testing set for **a** DLIGAND with a correlation coefficient of 0.526 and **b** DLIGAND2 with a correlation coefficient of 0.572. The solid line is from the regression fit

Figure 2 compares DLIGAND and DLIGAND2 in term of their ability for predicting protein–ligand binding affinity using the CASF-2013 dataset. DLIGAND2 achieves a higher Pearson correlation coefficient (PCC) (0.572) than DLIGAND (0.526). Table 1 further compares PCC values given by 29 other scoring functions. DLIGAND2 ranks the 9th among 30 scoring functions. The improvement of DLIGAND2 over DLIGAND was made without additional training. Interestingly, the top five scores (RF-Score-v2, ID-Score, ΔvinaRF_20_, AutoDock-hybrid and X-Score^HM^) were all trained directly for binding affinity prediction. The scoring function of Autodock Vina achieves a PCC of 0.56, which is lower than DLIGAND2 but higher than DLIGAND. According to the root mean square error (RMSE), DLIGAND2 (RMSE of 1.85) ranks the 10th after ChemPLP@GOLD (RMSE of 1.84), which is the best in all knowledge-based potential functions. The improvement in correlation coefficients is encouraging as DLIGAND2 was trained on protein structures only.

**Table 1:** Comparisons of 30 scoring functions on the CASF-2013 dataset

Function	PCC	RMSE	Description	Year
RF-Score-v2	0.803^a^	1.54	Machine learning	2014
ID-Score	0.753^b^	1.63	Descriptor-based and empirical	2013
ΔvinaRF_20_	0.686^c^	1.64	Machine learning	2016
AutoDockHybrid	0.64	n.a.	Force fields and machine learning	2016
X-Score^HM^	0.614	1.78	Empirical	2002
ΔSASA	0.606	1.79	Empirical	2014
ChemScore@SYBYL	0.592	1.82	Empirical	1998
ChemPLP@GOLD	0.579	1.84	Empirical	2009
*DLIGAND2*	*0.572*	*1.85*	*Knowledge-based*	*This paper*
SMoG2016	0.57^d^	1.68	Knowledge-based and empirical	2016
PLP1@DS	0.568	1.86	Empirical	2000
AutoDock Vina	0.563^e^	1.87	Knowledge-based and empirical	2010
G-Score@SYBYL	0.558	1.87	Energy-based	1997
ASP@GOLD	0.556	1.88	Statistical potential	2005
ASE@MOE	0.544	1.89	Empirical	n.a.
ChemScore@GOLD	0.536	1.90	Empirical	2003
*DLIGAND*	*0.526*	*1.92*	*Knowledge-based*	*2005*
D-Score@SYBYL	0.526	1.92	Energy-based	2001
Alpha-HB@MOE	0.511	1.94	Empirical	n.a.
LUDI3@DS	0.487	1.97	Empirical	1998
GoldScore@GOLD	0.483	1.97	Energy-based	1997
Affinity-dG@MOE	0.482	1.98	Empirical	n.a.
LigScore2@DS	0.456	2.02	Empirical	2005
GlideScore-SP	0.452	2.03	Energy-based	2006
SMoG2001	0.418	3.39	Knowledge-based	2001
Jain@DS	0.408	2.05	Empirical	2006
PMF@DS	0.364	2.11	Statistical potential	2006
GlideScore-XP	0.277	2.18	Energy-based	2004
London-dG@MOE	0.242	2.19	Empirical	n.a.
PMF@SYBYL	0.221	2.20	Statistical potential	1999

The results for 23 scoring functions were collected from Li [5], the results for RF-score-v2, ID-score, ΔvinaRF_20_ and SMoG2016 (labeled as ^a, b, c, d^) were collected from Ballester [57], Li [20], Wang [58] and Theau [31], separately, and the results for DLIGAND2, Autodock Vina, and DLIGAND were calculated with default options by ourselves

*n.a.* not available

#### Docking power

The docking power refers to whether a scoring function can correctly identify the native ligand poses from the predicted poses. Table 2 shows the evaluation results of docking power compared to the results by Li et al. [5] using the same docking sets in the CASF-2013 benchmark. DLIGAND2 achieves 14% improvement in success rate over DLIGAND in detecting native poses as the first ranked pose. Among all methods compared, DLIGAND2 has a moderate performance in term of success rates in ranking the native pose within top 1, 2, and 3 (at 45.1%, 61% and 75.4%, respectively). Nevertheless, DLIGAND2 ranks the second best in all knowledge-based/statistical potential scoring functions, behind ASP@GOLD, but better than PMF@SYBYL, PMF04@DS and PMF@DS. However, ASP@GOLD is not a pure statistical energy function but an empirical mix of a statistical potential with physical-based energetic terms in ChemScore@GOLD. Thus, DLIGAND2 has the best performance for parameter-free statistical potentials.

**Table 2:** Success rates for the evaluation of docking power ranked by top three poses

Scoring function	Success rates (%)		
	The top pose	Top two poses	Top three poses
ChemPLP@GOLD	81	86.7	89.7
ChemScore@GOLD	77.9	83.1	88.2
GlideScore-SP	78.5	85.6	87.7
ASP@GOLD	71.8	81.5	87.2
LigScore2@DS	76.9	84.1	86.7
PLP1@DS	77.4	84.1	86.2
PLP2@DS	74.4	81.5	86.2
Alpha-HB@MOE	75.4	82.6	86.2
GoldScore@GOLD	71.3	81	85.6
GlideScore-XP	74.4	82.6	85.6
LUDI1@DS	59	75.4	83.1
LUDI2@DS	65.6	75.4	81.5
LigScore1@DS	65.1	74.9	81
Affinity-dG@MOE	63.1	74.9	81
London-dG@MOE	59.5	73.8	78.5
X-Score^HM^	61	73.3	77.9
ChemScore@SYBYL	59.5	69.2	75.4
X-Score	59.5	69.2	75.4
*DLIGAND2*	*45.1*	*61*	*75.4*
X-Score^HP^	54.4	67.7	73.8
LUDI3@DS	48.7	65.1	72.8
GScore@SYBYL	45.1	61.5	72.3
X-Score^HS^	54.4	66.7	72.3
Jain@DS	48.2	62.1	70.8
PMF@SYBYL	51.8	60	66.7
PMF04@DS	51.8	62.6	66.2
ASE@MOE	51.3	60	63.6
PMF@DS	44.1	52.3	60
*DLIGAND*	*31.3*	*50.3*	*60.5*
dSAS	21.5	33.3	45.1
DScore@SYBYL	18.5	29.7	42.6

Results (excluding DLIGAND2 and DIGAND) cited from Li [5]. The RMSD value between one best-scored binding pose and the native binding pose is less than 2.0 Å

#### Ranking power

The ranking power of a scoring function refers to its ability to correctly rank binders of a given target protein by their predicted binding affinities based on the poses from the crystal structures and optimized structures. Table 3 compares DLIGAND and DLIGAND2 to the evaluation results of other scoring functions of ranking power collected by Li et al. [5]. A high-level success rate indicates a completely correct ranking of all members within each ligand cluster whereas a low-level success rate denotes ranking of the best as top 1 within a cluster. Again, DLIGAND2 has a small improvement over DLIGAND in high level success rates (1.6% on crystal structures and 3% on optimized structures) but identical in low-level success rates. Compared to other statistical potentials (PMF@DF, ASP@GOLD, PMF@SYBYL), DLIGAND2 has the highest high-level success rate in crystal and optimized structures and the highest low-level success rate in optimized structures but not in crystal structures. This suggests that DLIGAND2 is less sensitive to structural changes, compared to ASP@GOLD that has the large drop in low-level success rate from crystal to optimized structures. Empirical scoring functions such as X-Score and ChemScore@SYBYL have the best performance in this test.

**Table 3:** Success rates (%) for the evaluation of ranking power ranked by high-level results on optimized structures

Score function	Success rates (%) on crystal structures		Success rates (%) on optimized structures	
	High-level	Low-level	High-level	Low-level
X-Score^HM^	58.5	72.3	56.9	73.8
ChemScore@SYBYL	53.8	67.7	52.3	69.2
D-Score@SYBYL	49.2	63.1	52.3	63.1
LigScore1@DS	52.3	61.5	50.8	63.1
ΔSAS	49.2	67.7	50.8	69.2
*DLIGAND2*	*50.8*	*63.1*	*49.2*	*64.6*
PLP2@DS	55.4	72.3	47.7	67.7
Alpha-HB@MOE	52.3	66.2	47.7	64.6
ChemPLP@GOLD	58.5	72.3	46.2	61.5
G-Score@SYBYL	52.3	72.3	46.2	61.5
*DLIGAND*	*49.2*	*63.1*	*46.2*	*64.6*
PMF@DS	49.2	66.2	46.2	63.1
LUDI1@DS	52.3	69.2	44.6	66.2
Jain@DS	41.5	58.5	44.6	63.1
GoldScore@GOLD	55.4	76.9	43.1	66.2
ASE@MOE	40	64.6	43.1	63.1
London-dG@MOE	43.1	60	40	60
ASP@GOLD	47.7	72.3	38.5	60
Affinity-dG@MOE	53.8	66.2	36.9	50.8
ChemScore@GOLD	46.2	63.1	33.8	53.8
GlideScore-XP	35.4	47.7	32.3	46.2
PMF@SYBYL	43.1	61.5	30.8	53.8
GlideScore-SP	43.1	56.9	21.5	38.5

Results (excluding DLIGAND2 and DIGAND) cited from Li [5]

### Evaluation results on PDBbind data set

The above benchmark study is based on experimentally determined, protein–ligand complex structures. We further tested DLIGAND2’s ability to predict protein–ligand binding affinities by using predicted complex structures from docking. To remove random fluctuations, we generated 10 poses for each pair of protein and ligand by each docking method, and the highest score among 10 poses by each scoring function was used to represent the predicted binding affinity, respectively. As shown in Table 4, when scored by docking methods’ own scoring functions, AutoDock Vina yields the best correlation and lowest error with experimental values (PCC of 0.501 and RMSE of 1.75), followed by GalaxyDock (PCC of 0.487 and RMSE of 1.75) and iDock (PCC of 0.485 and RMSE of 1.75). rDock and UCSF dock have PCC < 0.2 and RMSE > 1.95. Low performance by rDOCK and UCSF was consistent with a previous study [4].

**Table 4:** Pearson correlation coefficients and root mean squared error between experimental binding affinity and binding affinity predicted by DLIGAND, DLIGAND2, and X-Score using docking poses generated by eight docking programs along with the results from the docking programs

Docking program	Pearson correlation coefficient				Root mean squared error			
	Self	DLIGAND	DLIGAND2	X-Score	Self	DLIGAND	DLIGAND2	X-Score
AutoDock	0.404	0.465	0.537	0.547	1.91	1.77	1.69	1.68
AutoDock Vina	0.501	0.459	0.519	0.536	1.74	1.78	1.72	1.69
rDock	0.102	0.463	0.535	0.507	2.01	1.78	1.70	1.76
LeDock	0.426	0.457	0.532	0.54	1.82	1.78	1.69	1.69
UCSF DOCK	0.195	0.427	0.498	0.488	1.97	1.81	1.74	1.76
iDock	0.485	0.461	0.522	0.54	1.75	1.78	1.71	1.69
GalaxyDock	0.487	0.464	0.537	0.532	1.75	1.78	1.69	1.71
iGEMDOCK	0.384	0.444	0.501	0.502	1.85	1.80	1.76	1.82
Average	0.373	0.455	0.523	0.524	1.85	1.79	1.71	1.73

When re-assessed by DLIGAND2, the PCCs of predicted binding affinity consistently improve over all eight docking methods to the levels from 0.498 to 0.537 with an average of 0.523, and the RMSE from 1.69 to 1.76 with an average of 1.71. This indicates the main bottleneck of current docking method is the scoring function, as also disclosed in the previous study [4]. By comparison, DLIGAND can improve PCC values for five docking programs but decrease PCC values for 3 others with an average PCC of 0.455 and RMSE of 1.79, which are 13% lower and 4.7% higher than DLIGAND2, respectively. On the basis of average value, X-Score has a performance comparable to DLIGAND2 in PCC but a slightly higher error in RMSE. It should be noted that X-Score was trained on the complex structures homologous to the CASF-2013 benchmark dataset used here, whereas DLIGNAD2 was trained only by independent monomer structures. We also noted that DLIGAND2 is about 5 times faster than X-Score, which takes 2.7 and 13.3 h, respectively to complete this dataset (a total of 40,440 docking poses) by one CPU core of the Intel E5-2692V2 (2.2 GHz). Here, we did not compare to RF-Score (including RF-Score-v4 [59]), ΔvinaRF_20_ and ID-Score because they were trained on the PBDbind refined set.

### Evaluation results on DUD-E data set

The DUD-E dataset is used to examine the ability to separate true ligands from decoys, a practically important problem in virtual screening. Here, we employed the DUD-E dataset to evaluate the screening power of scoring functions. The performance of DLIGAND and DLIGAND2 is compared to those of three top ranked scoring functions in the CASF-2013 benchmark (ID-Score, ΔvinaRF_20_, and X-Score) using the poses generated by AutoDock Vina.

As shown in the Table 5 (The detailed data can be found in Additional file 4: Table S4), DLIGAND2 achieved the best performance with an average logAUC of 10.14% and enrichment factors of 6.67 for EF_1%_. DLIGAND2 achieved an average EF_1%_ of 30% higher than Autodock Vina, 52% and 64% higher than DLIGAND and X-Score, separately, and above 3 times higher than ID-score. The logAUC and enrichment factors of all targets are detailed in Additional file 4: Table S4. Notably, Autodock Vina ranks the 2nd by LogAUC and the first on EF_5%_ and EF_10%_, with EF_1%_ of 26% and 86% higher than those by X-Score, and ID-Score despite the fact that they can provide higher correlation coefficients than Autodock Vina to experimental binding affinities in the CASF-2013 dataset. This is likely because ID-Score and X-Score were all trained by the PDBbind dataset that are homologous to CASF-2013 dataset. The over-training issues in empirical or machining learning based scoring functions have also been observed in several previous studies [23]. The improvement of DLIGAND2 relative to Autodock Vina is more consistent in this independent test. As for RF-Score, the general version (RF-Score v3) for predicting binding affinity doesn’t achieve a good performance with 5.42 for EF_1%_ [53], ranking even behind DLIGAND. Although RF-Score-VS version specifically trained based on DUD-E was reported to achieve EF_1%_ values up to 38.96 [53], the per-target cross validation tends to have an over-estimate due to protein homologs between training and test sets [60]. We will employ an external DEKOIS 2.0 dataset to evaluate DLIGAND2 and RF-Score-VS separately below.

**Table 5:** The performance of six scoring functions on the DUD-E dataset

Scoring functions	LogAUC(%)	EF_1%_	EF_5%_	EF_10%_
DLIGAND2	*10.14*	*6.67*	3.31	2.55
AutoDock Vina	9.96	5.12	*3.41*	*2.60*
ΔvinaRF20	9.00	6.38	*3.41*	2.58
DLIGAND	7.61	4.40	2.74	2.23
X-Score^HM^	7.25	4.06	2.68	2.19
ID-Score	2.47	1.61	1.42	1.36

The highest values in each column are labeled italics

To further compare the performance of each scoring function for different protein categories, 102 targets of DUD-E dataset are separated into eight categories and evaluated by the average EF_1%_ as shown in Table 6. DLIGAND2 has the highest values of EF_1%_ in Cytochrome P450, GPCR, Kinase, and Protease. Especially in the category of GPCR and Kinase, DLIGAND2 has obvious advantages compared with other scoring functions by 2.03 times and 1.42 times better than the second ranked methods (ΔvinaRF_20_ and DLIGAND), respectively. By comparison, AutoDock Vina performs the best in the ion channel, and is far superior to DLIGAND. The scoring function ΔvinaRF_20_ performs the best in miscellaneous, nuclear receptors and other enzymes. DLIGAND2 doesn’t perform well in targets of kinesin-like protein 1 (KIFF11, miscellaneous) and poly (ADP-ribose) polymerase-1 (PARP1, other enzymes), likely because their binding ligand contains halogen and phosphate elements that don’t appear in training protein chains. Currently, DLIGAND2 simply treats phosphate elements equivalent to the sulfate atom type. This issue may be solved in future study by including additional ligand atoms from protein–ligand complex structures.

**Table 6:** Enrichment factor values (EF_1%_) by DLIGAND2, AutoDock Vina, ΔvinaRF_20_, DLIGAND, X-Score^HM^, ID-Score on eight protein categories

	DLIGAND2	Vina	ΔvinaRF_20_	DLIGAND	X-Score^HM^	ID-Score
Cytochrome P450	*6.48*	1.93	3.77	5.10	3.59	0.56
GPCR	*8.46*	2.48	4.17	3.79	1.56	1.49
Ion channel	0.84	*4.47*	3.48	0.51	0.00	0.83
Kinase	*10.01*	6.10	7.50	4.41	5.93	1.04
Miscellaneous	6.89	5.65	*7.82*	6.63	4.46	5.36
Nuclear receptors	5.42	9.14	*9.57*	4.57	4.01	1.69
Other enzymes	3.23	3.88	*4.76*	2.52	2.64	1.21
Protease	*10.16*	4.63	6.99	8.65	5.58	2.57
Average	*6.43*	4.79	6.01	4.52	3.47	1.84

Italic fonts highlight the highest value in each category

**Fig. 3:**
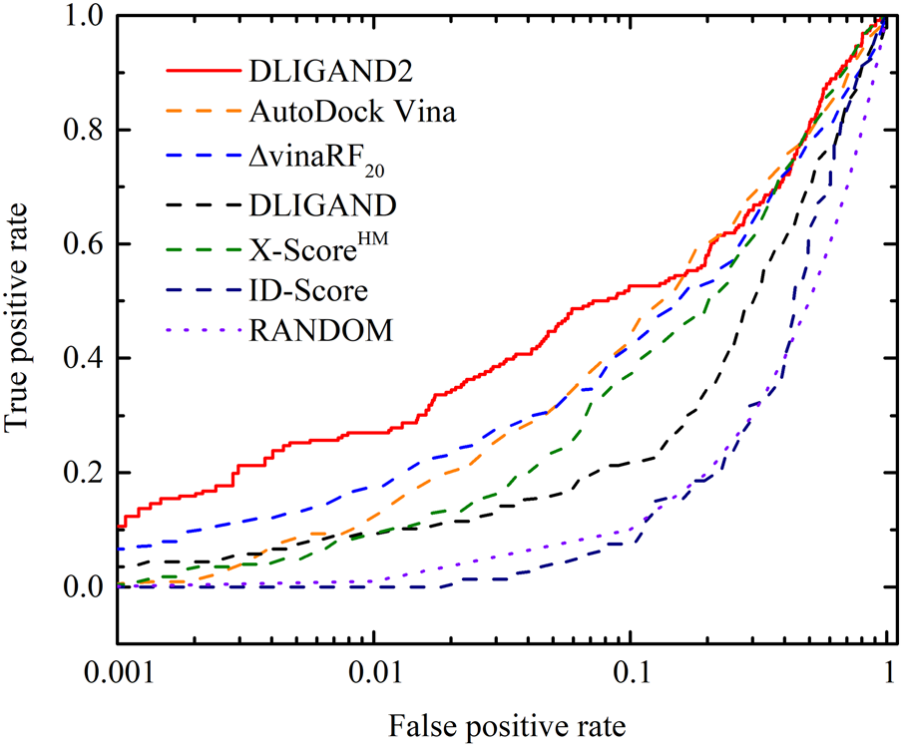
Receiver operating characteristic (ROC) curves for the target PTN1 protein by different scoring methods

Among the best examples of DLIGAND2 performance, we plotted the receiver operating characteristic (ROC) for the case of PTN1 protein (protein-tyrosine phosphatase 1B). As shown in Fig. 3, DLIGAND2 has the highest area under the curve (AUC) of 0.769, followed by AutoDock Vina (0.75), X-Score (0.729) and DLIGAND (0.639). The differences are more significant at lower false positive rate, the most important region for virtual screening. Indeed, the EF_1%_ are 28.89, 9.12, 18.32, 9.33 and 9.33 for DLIGAND2, Autodock Vina, ΔvinaRF20, DLIGAND and X-Score, respectively. The AUC of ID-Score is 0.553, close to 0.5 by the random selection.

### Evaluation results on DEKOIS 2.0 data set

**Fig. 4:**
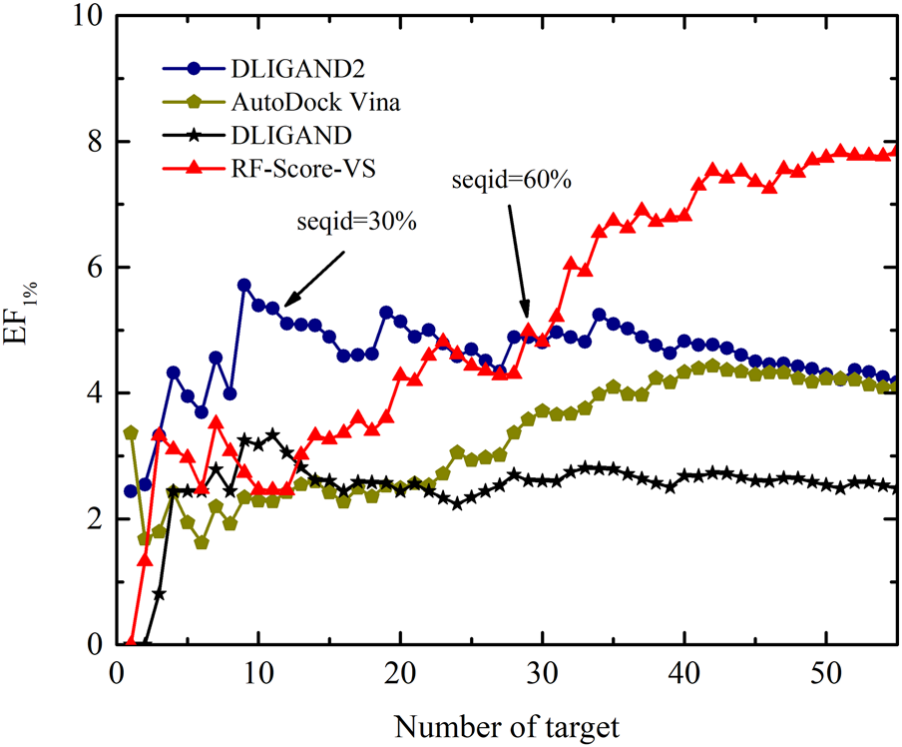
The average EF_1%_ in the DEKOIS 2.0 benchmark over the number of targets sorted according to their increasing sequence identity (seqid) by blastpgp from the DUD-E targets

To compare with the latest RF-Score-VS v2 (https://github.com/oddt/oddt) scoring function that was trained on the DUD-E, we have compiled a new dataset from the DEKOIS 2.0 benchmark, with all targets sorted according to their sequence identity to the DUD-E targets according to the blastpgp. Figure 4 plots the average enrichment factor (EF_1%_) as a function of the number of targets sorted according to sequence identity. The average EF_1%_ for RF-Score-vs increases as the sequence identity increases, suggesting the performance of RF-Score-VS v2 is strongly depending on similarity to its training set. AutoDock Vina also has some dependence on similarity to DUD-E targets. By comparison, DLIGAND and DLIGAND2 have the least dependence except when the number of targets is low (< 10) likely due to natural fluctuations. DLIGAND2 has the highest performance when homologous targets are excluded for sequence identity less then 30% with an average EF_1%_ at 5.72, compared to 2.34 by AutoDock Vina, 2.73 by RF-Score-VS and 3.25 by DLIGAND.

## Conclusion

We have developed a new knowledge-based scoring function DLIGAND2 by extending to 167 atom types for protein atoms from 13 types in the original DLIGAND. Residue-specific atom types for proteins allow a more accurate description of the interaction of a ligand atom with different residues. To ensure sufficient statistics, DLIGAND2 is based on an updated non-redundant dataset of 12,450 protein chains, 62 times bigger than the dataset (195 structures) used in the original DLIGAND.

DLIGAND2 consistently improves over DLIGAND in binding affinity prediction using either native or docking-predicted complex structures. The improvement in Pearson correlation coefficient is 8.7% for the CASF-2013 dataset by using native complex structures and 15% for the PDBbind dataset by using predicted complex structures. In addition, DLIGAND2 has significantly higher enrichment than DLIGAND in discriminating true ligands from decoys using the DUD-E dataset according to re-ranking of docked structures. These results suggest the usefulness of expanding protein atomic types in generating the DLIGAND 2 statistical potential.

DLIGAND2 is the best knowledge-based energy score but not as accurate as a few empirical (X-Score) or machine-learning based (RF-Score-v2 and ID-Score) scores trained by CASF-2013 or PDBbind. The X-Score and ID-Score methods outperform Autodock vina in the CASF-2013 and PDBbind, but they all have lower performance in decoy discrimination, a practically more important problem. We have also shown that the performance of RF-score-vs strongly depends on the sequence identity of the target protein to the dataset for training the method. Though RF-score-vs was reported to perform well in the DUD-E that includes many homologous proteins to its training set, it doesn’t perform well on protein targets that are not homologous to its training set. By comparison, DLIGAND2 was derived from only protein monomer structures, ensuring a balanced performance for all targets. Considering the simplicity and fast computation, DLIGAND2 will be useful for re-scoring after docking, or being included as a term for other scoring functions.

## Additional files

**Additional file 1: Table S1.** Showed 13 mol2 atom types mapped to 167 residue-specific atom types for protein atoms.

**Additional file 2: Table S2.** It contained the list of the 4044 complexes collected from PDBind refined data set.

**Additional file 3: Table S3.** The EF_1%_ values of four scoring functions on DEKOIS 2.0 data set are shown.

**Additional file 4: Table S4.** The logAUC and EF (1%, 5% and 10%) values of six scoring functions on the DUD-E dataset are listed.
